# Human Hepatocytes and Differentiated Adult-Derived Human Liver Stem/Progenitor Cells Display *In Vitro* Immunosuppressive Properties Mediated, at Least in Part, through the Nonclassical HLA Class I Molecule HLA-G

**DOI:** 10.1155/2019/8250584

**Published:** 2019-09-12

**Authors:** Catherine A. Lombard, Gwenaëlle Sana, Joël LeMaoult, Mehdi Najar, Joachim Ravau, Floriane André, Fatima Bouhtit, Marina Daouya, Maria Loustau, Mustapha Najimi, Laurence Lagneaux, Edgardo D. Carosella, Etienne M. Sokal

**Affiliations:** ^1^Laboratory of Pediatric Hepatology and Cell Therapy, Institut de Recherche Expérimentale et Clinique, Université Catholique de Louvain & Cliniques Universitaires Saint-Luc, Avenue Hippocrate 10, 1200 Brussels, Belgium; ^2^Service de Recherches en Hémato-Immunologie, CEA-DSV-DRM, Hôpital Saint-Louis, IUH, Avenue Claude Vellefaux 1, 75010 Paris, France; ^3^Osteoarthritis Research Unit, Department of Medicine, University of Montreal Hospital Research Center (CRCHUM), 900 rue Saint-Denis, R11.424, Montreal, QC, Canada H2X 0A9; ^4^Laboratory of Clinical Cell Therapy, Institut Jules Bordet, Université Libre de Bruxelles (ULB), Campus Erasme, Brussels, Belgium

## Abstract

One of the main challenges in liver cell therapy (LCT) is the induction of a tolerogenic microenvironment to promote graft acceptance in the recipient. Little is known about the immunomodulatory potential of the hepatic cells used in liver cell therapy. In this work, we wanted to evaluate the immunosuppressive properties of human hepatocytes and adult-derived human liver stem/progenitor cells (ADHLSCs), as well as the potential involvement of the immunomodulatory molecule HLA-G. We demonstrated that both cell types were capable of inhibiting the proliferative response of PBMCs to an allogenic stimulus and that the immune inhibitory potential of ADHLSCs, although lower than that of hepatocytes, increased after hepatogenic differentiation. We demonstrated that liver cells express HLA-G and that the immune inhibition pattern was clearly associated to its expression. Interestingly, HLA-G expression increased after the third step of differentiation, wherein oncostatin M (OSM) was added. A 48 hr treatment with OSM was sufficient to induce HLA-G expression in ADHLSCs and result in immune inhibition. Surprisingly, blocking HLA-G partially reversed the immune inhibition mediated by hepatocytes and differentiated ADHLSCs, but not that of undifferentiated ADHLSCs, suggesting that additional immune inhibitory mechanisms may be used by these cells. In conclusion, we demonstrated that both hepatocytes and ADHLSCs present immunomodulatory properties mediated, at least in part, through HLA-G, which can be upregulated following hepatogenic differentiation or liver cell pretreatment with OSM. These observations open up new perspectives for the induction of tolerance following LCT and for potential therapeutic applications of these liver cells.

## 1. Introduction

One of the main challenges in cell therapy is the induction of a tolerogenic microenvironment which would help promote graft acceptance in the recipient. The level of tolerance achieved closely depends on the immunomodulatory properties of the transplanted cells.

In the field of liver cell therapy, hepatocyte transplantation has already demonstrated its safety and medium-term success in correcting metabolic disorders [1]. However, because of limited hepatocyte availability and viability, other cell sources are under development for liver cell transplantation, including adult-derived human liver stem/progenitor cells (ADHLSCs) [1]. These cells, characterized by a hepatic origin and a mesenchymal phenotype, present the advantage of a high proliferative capacity and the ability to differentiate into functional hepatocyte-like cells *in vitro* and *in vivo* [2–4].

Previous studies have suggested that both cell types could potentially present an immunotolerogenic capacity, owing to their hepatic and/or mesenchymal origin. Indeed, the liver is widely considered as an immunoprivileged organ that can favour the induction of immunologic hyporesponsiveness or even tolerance [5]. Liver tolerance has been highlighted by several lines of evidence, such as the relatively low occurrence of T-cell-mediated rejection in liver transplant recipients and, in some cases, the acceptance of liver grafts despite the absence of an immunosuppressive therapy, as well as the demonstration of the liver transplant's ability to improve the acceptance of other grafted organs [6, 7]. Similarly, mesenchymal stem cells (MSCs) of various origins have been known for their immunomodulatory properties (reviewed in [8]), supporting their use for different immunotherapy indications [9]. *In vitro*, bone marrow-derived MSCs (BM-MSCs) have been shown to modulate the function of various immune cell populations, including dendritic cells and T cells. *In vivo*, BM-MSCs have been used to prevent graft-versus-host disease following hematopoietic cell transplantation. Several soluble factors have emerged as key molecules in MSCs' immunomodulatory properties, including HLA-G, IL-10, IDO, PGE_2_, INOS, and TGF-*β* [8]. Among these immunosuppressive factors, HLA-G has been described to play a role in both the induction of tolerance following allogeneic transplantation and in MSC-mediated immunosuppression [10, 11].

Human leukocyte antigen (HLA)-G is a nonclassical MHC class I molecule characterized by a very low polymorphism. HLA-G can be expressed as seven isoforms (four membrane-bound proteins: HLA-G1, HLA-G2, HLA-G3, and HLA-G4; and three soluble proteins: HLA-G5, HLA-G6, and HLA-G7) resulting from the alternative splicing of the HLA-G primary transcript [12, 13]. HLA-G1 and HLA-G5 share a common extracellular structure comprising the same heavy chain bound to *β*2-microglobulin [14]. In addition, HLA-G1 can be found in a soluble form following cleavage from the cell membrane [14]. HLA-G was initially described in cytotrophoblasts, where it plays a major role in semiallogeneic foetus tolerance [15, 16]. Its expression remains marginal and restricted to a small number of healthy tissues such as the thymus, cornea, erythroid precursors, and blood cells [14, 17–19]. In the context of transplantation, several *in vivo* studies have suggested that HLA-G molecules are involved in the induction of allogeneic graft tolerance. Indeed, the expression of HLA-G on graft biopsies of heart-, liver-, kidney-, or liver-kidney-transplanted patients has been correlated with a reduced incidence of acute and/or chronic rejection [20–22]. Moreover, an increased blood level of HLA-G molecules has been detected in patients with a reduced incidence of acute rejection after allograft transplantation [22–26]. Further *in vitro* experiments have supported the immunosuppressive role of HLA-G, demonstrating its strong faculty to inhibit various immune functions such as NK cell and T cell cytolysis activities, allogeneic T cell proliferation, and dendritic cell maturation and function [27–31]. The induction of regulatory T cells by HLA-G was also described [32, 33]. These inhibitory functions of HLA-G are mediated through its interactions with immunoglobulin-like transcript 2 (ILT-2) and 4 (ILT-4) receptors and killer immunoglobulin-like receptor 2DL4 (KIR2DL4) [34].

ADHLSCs under proliferative conditions have previously been shown to suppress the proliferative response of T cells to a mitogenic stimulus [35]. In addition, they have been shown to express HLA-G [35]. However, the direct link between HLA-G expression and immune inhibitory capacity has not been demonstrated. Furthermore, hepatocytes and ADHLSCs differentiated into hepatocyte-like cells had not been investigated. In this article, we demonstrate that both human hepatocytes and ADHLSCs present *in vitro* immune inhibitory properties. In addition, we evaluate the expression of HLA-G on these liver cells and its involvement in their immune inhibitory effect.

## 2. Materials and Methods

### 2.1. Cell Isolation and Culture

#### 2.1.1. Human Hepatocyte Isolation Procedure

The present study was approved by the local ethics committee. Written informed consent was obtained from each individual before blood sampling for PBMC isolation or from next of kin for cadaveric liver donors. Human hepatocytes (Hep) were isolated from donor livers (Table 1) using the two-step collagenase perfusion technique. Each donor was used individually. Cell viability was evaluated by the Trypan blue (Thermo Fisher Scientific, Merelbeke, Belgium) dye exclusion method. The cells were then either used fresh or cryopreserved for later use. Liver isolation and hepatocyte cryopreservation/thawing procedures were previously published in detail [2, 36]. Human hepatocytes were cultured on collagen I-coated plates (Becton Dickinson, Erembodegem, Belgium) using Williams' E medium (Thermo Fisher Scientific) supplemented with 10% fetal calf serum (FCS) (HyClone, Erembodegem, Belgium), 25 ng/ml epidermal growth factor (EGF) (PeproTech, London, UK), 10 *μ*g/ml insulin (Eli Lilly, Brussels, Belgium), and 1% of Penicillin/Streptomycin (Thermo Fisher Scientific).

#### 2.1.2. ADHLSC Preparation and Culture

ADHLSCs emerged from the primary culture of the parenchymal fraction obtained following a two-step collagenase digestion of healthy human livers (Table 1), as previously described [2, 37]. The cells were cultured in CellBIND T175 cm^2^ flasks (VWR, Leuven, Belgium) using DMEM medium containing a high glucose concentration (4.5 g/l) (Thermo Fisher Scientific) and supplemented with 10% FCS and 1% of Penicillin/Streptomycin (complete DMEM medium) at 37°C in a fully humidified atmosphere under 5% CO_2_. Upon reaching 80% confluence, the cells were lifted using 0.05% trypsin-EDTA (Thermo Fisher Scientific) and seeded at 5000 cells/cm^2^.The cells were used at passage 5 or 6, as these are the passages currently used for cell therapy in the clinic. Cell viability was evaluated by the Trypan blue dye exclusion method.

#### 2.1.3. PBMC Isolation

PBMCs were isolated from the buffy coat leukocytes of healthy volunteers (Belgian Red Cross, Namur Blood Center, Namur, Belgium) by density gradient centrifugation using Ficoll-Paque (GE Healthcare, Diegem, Belgium). Briefly, total blood was overlaid onto a double volume of Ficoll-Paque and centrifuged at 900 g at room temperature (RT) for 20 min with no break. The white blood cell ring was collected, and PBMCs were enriched by 3 successive washes in HBSS (Thermo Fisher Scientific) at 860, 690, and 200 g. Finally, the PBMCs were resuspended in RPMI-1640 (Thermo Fisher Scientific) supplemented with 1% of Penicillin/Streptomycin, 2 mM L-glutamine (Thermo Fisher Scientific), 1% nonessential amino acids (Thermo Fisher Scientific), and 10% heat-inactivated FCS (complete RPMI medium). Cell viability, as estimated by Trypan blue exclusion, was >95%. Cells were cryopreserved in FCS containing 10% DMSO and defrosted as needed. Thawed cells were placed in warm complete RPMI medium, centrifuged at 260 g for 15 min, and treated with 100 IU/ml of DNase I (Sigma Aldrich, Overijse, Belgium) in complete RPMI medium for 15 min, followed by 2 washes in HBSS. The cells were then allowed to rest overnight in complete RPMI, before use [38].

#### 2.1.4. Hepatogenic Differentiation

ADHLSCs at passage 5 or 6 were seeded at a density of 1 × 10^4^ cells/cm^2^ onto collagen I-coated 175 cm^2^ flasks in complete DMEM medium. Twenty-four hours later, the culture medium was switched to IMDM (Thermo Fisher Scientific). Cells were then subjected to a four-step differentiation protocol as previously described [39]. First, cells were incubated for 2 days with IMDM containing 20 ng/ml epidermal growth factor (EGF) (PeproTech) and 10 ng/ml basic fibroblast growth factor (bFGF) (PeproTech). Then, the cells were incubated for 10 days with IMDM containing 20 ng/ml hepatocyte growth factor (HGF) (PeproTech), 10 ng/ml bFGF, nicotinamide 0.61 g/l (Sigma Aldrich), and 1% insulin-transferrin-selenium (ITS) (Invitrogen) premix. Next, the cells were incubated for 10 days with IMDM containing 20 ng/ml HGF, 20 ng/ml oncostatin M (OSM) (PeproTech), 0.61 g/l nicotinamide, and 1% ITS premix. Finally, the cells were treated with IMDM containing 20 ng/ml OSM, 1 *μ*M dexamethasone (Sigma Aldrich), and 1% ITS premix for 10 days. For each step, the medium was changed every three days. Negative controls were performed using IMDM supplemented with 1% FCS and 1% of Penicillin/Streptomycin. Cells were harvested either after each step or at the end of the differentiation protocol and used for MLR, RT-PCR, fluorescence microscopy, or flow cytometry analysis.

#### 2.1.5. OSM and INF-*γ* Treatment

ADHLSCs were seeded at a density of 1 × 10^4^ cells/cm^2^ on a collagen I-coated 175 cm^2^ flask in complete DMEM medium. The next day, the cells were incubated in IMDM supplemented with 20 ng/ml OSM for 48 hours, using IMDM supplemented with 1% FCS as a control. ADHLSCs treated with IFN-*γ* (1000 UI/ml) were used as a positive control for HLA-G upregulation. The cells were then harvested and used for further analysis.

### 2.2. Real-Time Quantitative Reverse Transcription-Polymerase Chain Reaction (RT-PCR)

Total RNA was extracted from liver-derived cells using the TriPure isolation reagent (Sigma Aldrich) as previously described [37]. Following DNAse I treatment, cDNA was generated from 5 *μ*g of RNA using the ThermoScript RT-PCR system according to the manufacturer's instructions (Thermo Fisher Scientific) [37]. RT-PCR for HLA-G primary transcripts was performed using a StepOnePlus thermocycler (Applied Biosystems, Thermo Fisher Scientific) as previously described [40]. Briefly, PCR was carried out for 40 amplification rounds in the presence of TaqMan Universal PCR Master Mix (Thermo Fisher Scientific), using the predeveloped TaqMan assay reagent GAPDH as internal control. A HLA-G-specific probe located in exon 5 (200 nM: 5′-CACTGGAGCTGCGGTCGCTGCT-3′; 6-carboxyfluoresceine (FAM) reporter, and TAMRA quencher) and HLA-G-specific primers (300 nM: forward 5′-CTGGTTGTCCTTGCAGCTGTAG-3′; reverse 5′-CCTTTTCAATCTGAGCTCTTCTTTCT-3′) (Thermo Fisher Scientific), which amplify all alternative HLA-G forms, were used. Quantification relative to the human placental choriocarcinoma cell line JEG-3 was performed using the comparative *C*_*T*_ method: Δ*C*_*T*_ = *C*_*T* HLA−G_ − *C*_*T* GAPDH_; ΔΔ*C*_*T*_ = Δ*C*_*T* sample_ − Δ*C*_*T* JEG−3_; relative HLA-G expression = 2^−ΔΔCT^.

### 2.3. Fluorescence Microscopy Analysis

Human hepatocytes (5 × 10^4^ cells) were resuspended in D-PBS (Westburg, Leusden, the Netherlands) and deposited onto cytospin slides by centrifugation at 700 g for 5 min. ADHLSCs were cultured in collagen I-coated Labtek chambers (Thermo Fisher Scientific). Cells were fixed with 4% paraformaldehyde (Sigma Aldrich) for 10 min. After washing, cells were permeabilized with PBS-Triton X-100 0.1% (Sigma Aldrich). Cells were washed and incubated for 45 min at RT in PBS-BSA 3% (Sigma Aldrich) with primary mouse anti-human monoclonal HLA-G IgG1, clone 4H84, recognizing all HLA-G forms (5 *μ*g/ml) or 5A6, recognizing the HLA-G5 and 6 soluble forms (5 *μ*g/ml) (Gentaur, Kampenhout, Belgium). After washing, cells were exposed for 45 min to secondary antibody Alexa Fluor 488-conjugated goat anti-mouse IgG (5 *μ*g/ml) (Thermo Fisher Scientific). Samples were rinsed and mounted in VECTASHIELD medium with DAPI (Labconsult, Brussels, Belgium) for analysis. Negative experimental controls were performed with the corresponding isotype mouse anti-human IgG1. Images were acquired by fluorescence microscopy on an Axio Imager and ApoTome (Zeiss, Zaventem, Belgium) (IREC 2IP platform, UCLouvain).

### 2.4. Flow Cytometry

The expression of HLA-G by human liver cells was evaluated by flow cytometry using PE-anti-HLA-G1/-G5 IgG1 (clone MEM-G9; Gentaur), as previously described [41].

Cells were washed with PBS containing 0.5% BSA and 2 mM EDTA (MACS Buffer) (Thermo Fisher Scientific), and cell surface staining was performed by incubating the cells for 30 minutes at RT with the antibody or the corresponding isotype. Intracytoplasmic staining was performed by incubating the cells in FIX & PERM reagent (Thermo Fisher Scientific) according to the manufacturer's instructions. Cells were washed with MACS buffer and incubated with the antibody or the corresponding isotype for 30 minutes at RT. After staining, the cells were washed and analyzed on a MACSQuant analyzer (Miltenyi Biotec, Leiden, the Netherlands). The human placental Jeg-3 and JAR choriocarcinoma cell lines were used as positive and negative controls, respectively.

### 2.5. Mixed Lymphocyte Reaction

In order to assess the immunosuppressive potential of liver cells, irradiated (25 Gray) human hepatocytes or ADHLSCs were seeded at densities of 50000, 25000, and 12500 cells per well in 96-well CellBIND plates and allowed to adhere overnight. The next day, isolated PBMCs (10^5^ cells/well) were used as responder cells and cocultured in a mixed lymphocyte reaction (MLR) with 10^5^*γ*-irradiated (25 Gray) allogeneic stimulator PBMCs, in the presence or absence of liver cells, in 200 *μ*l of complete RPMI medium. Where specified, neutralizing antibody directed against HLA-G (clone 87G) was added at a concentration of 20 *μ*g/ml on day 0 and day 3, using the corresponding IgG2a isotype as a negative control, as previously described [10]. On day 6, cells were pulsed with [^3^H]-thymidine (1 *μ*CI/well; MP Biomedicals, Irvine, CA), and after 18 to 24 hours, the allogeneic proliferative response was evaluated by *β*-scintillation counting with a TopCount NXT microplate scintillation and luminescence counter (PerkinElmer, Zaventem, Belgium). For each experiment, the proliferative response observed for the MLR alone was assigned a value of 100%. Each result was compared to the proliferative response of the MLR.

### 2.6. Quantification of sHLA-G in Culture Supernatants

For the analysis of HLA-G secretion, hepatocytes and undifferentiated and differentiated ADHLSCs were seeded at a density of 100000 cells/well in collagen-coated 6-well plates in their respective medium and were allowed to adhere overnight. The next day, the medium was changed to phenol-free and serum-free media. Supernatants were collected after 24 hours, centrifuged to remove cell debris, and then stored at -80°C until use. Cells were trypsinized and enumerated using the Trypan blue dye exclusion method to normalize the results to an equivalent cell number. Soluble HLA-G was quantified in 50 *μ*l of culture supernatant using a Luminex assay as described by Rebmann et al. [42]. Recombinant HLA-G5-B2M protein was used as a standard ranging from 200 ng/ml to 0.39 ng/ml [43]. The results are expressed as pg/ml/100000 cells/24 hours.

### 2.7. Statistical Analysis

Results are expressed as mean ± SEM. Statistical analyses were performed using the GraphPad Prism 7.04 software (GraphPad software, San Diego, USA). Single comparisons were performed using unpaired two-tailed Student's *t*-test. Multiple comparisons were performed using the one-way ANOVA analysis with the Dunnett or Tukey post hoc test. Results were considered statistically significant when *p* < 0.05 (^∗^*p* < 0.05 and ^∗∗∗∗^*p* < 0.0001).

## 3. Results

### 3.1. Characterization of Adult-Derived Human Liver Stem/Progenitor Cells

ADHLSCs have been extensively characterized [2, 4, 37, 39, 44]. The ADHLSCs used in this study presented the typical characteristics previously described by our group: expression of CD73, CD90 CD105, HLA-ABC, and albumin; absence of CD45, CD40, CD86, HLA-DR, cytokeratin 7 (CK7), and CK19; and the capacity to differentiate into hepatocyte-like cells, characterized by round- or polygonal-shaped cells reduced in size, containing cytoplasmic granulations and central nucleus with prominent nucleolus, when subjected to a specific differentiation protocol (data not shown).

#### 3.1.1. Human Hepatocytes and Liver Progenitor Cells Possess Immune Inhibitory Properties In Vitro

**Figure 1:**
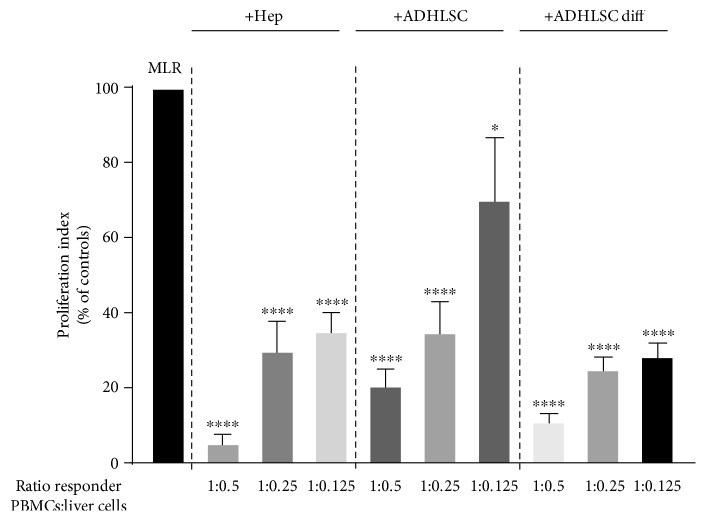
The in vitro immune inhibitory properties of human liver cells. Hepatocytes, undifferentiated ADHLSCs, or differentiated ADHLSCs were added as third-party cells to MLR cultures for 7 days. Liver cells were added to the MLR at the indicated MLR : cells ratios of 1 : 0.5, 1 : 0.25, and 1 : 0125. The proliferation of allogeneic responder PBMCs was evaluated by the [3H]-thymidine incorporation method. The proliferation obtained in MLR was arbitrarily assigned a value of 100% and used as a reference to calculate the percentage of proliferation obtained with the other conditions. The bars represent the mean + SEM of at least 3 independent experiments. The data were analyzed using the one-way ANOVA test followed by the Dunnett post hoc test for multiple comparisons of the MLR group to the other treatment groups. ^∗^*p* < 0.05 and ^∗∗∗∗^*p* < 0.0001.

We first investigated the immune inhibitory potential of hepatocytes and ADHLSCs *in vitro*. Using the [^3^H]–thymidine incorporation assay, we assessed the effect of these liver cells on the proliferative response of PBMCs in a mixed lymphocyte reaction (MLR). As shown in Figure 1, we found that hepatocytes and undifferentiated ADHLSCs were both capable of preventing the proliferative response of PBMCs to an allogeneic stimulus in a dose-related manner, although ADHLSCs exhibited a lower immune inhibitory effect than the hepatocytes. Interestingly, following hepatogenic differentiation *in vitro*, the level of immune inhibition induced by ADHLSCs reached a level similar to that seen with the hepatocytes.

#### 3.1.2. Human Hepatocytes and Liver Progenitor Cells Expressed HLA-G Molecules

Because HLA-G has emerged as a key molecule in MSC-mediated immune inhibition and we have previously demonstrated that it is expressed by ADHLSCs under proliferative conditions, we investigated its expression by human hepatocytes and ADHLSCs subjected to a hepatogenic differentiation protocol, using cells maintained in medium with 1% FCS as an undifferentiated control.

**Figure 2:**
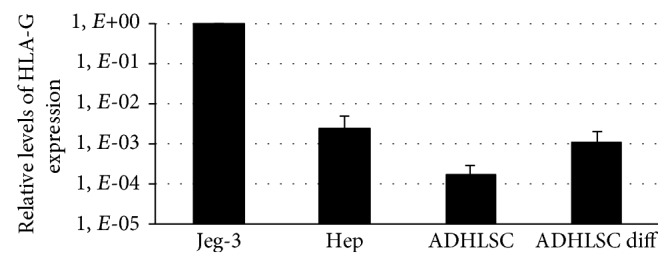
Analysis of HLA-G mRNA expression by human liver cells. Total RNA was extracted from hepatocytes as well as undifferentiated and differentiated ADHLSCs, and cDNA was synthesized and used in a real-time RT-PCR assay for primary HLA-G transcripts. The choriocarcinoma cell line Jeg-3 was used as a positive control of HLA-G mRNA expression. Results are expressed as the mean ± SEM of three independent experiments and are depicted as relative quantities of the HLA-G transcript compared to that found in Jeg-3 (arbitrarily assigned a value of 1).

We first evaluated HLA-G expression at the mRNA level by real-time RT-PCR using Jeg-3, a choriocarcinoma cell line expressing high levels of HLA-G, as a positive control. As shown in Figure 2, both human hepatocytes and ADHLSCs presented a detectable level of HLA-G transcripts. However, expression levels were slightly lower in ADHLSCs than in hepatocytes. Interestingly, after differentiation of ADHLSCs into hepatocyte-like cells, the HLA-G mRNA expression had a tendency to increase to a level similar to that of mature hepatocytes. It has to be noted that the pattern of HLA-G expression seemed to follow the immune inhibitory capacity of hepatocytes and ADHLSCs depicted in Figure 1.

**Figure 3:**
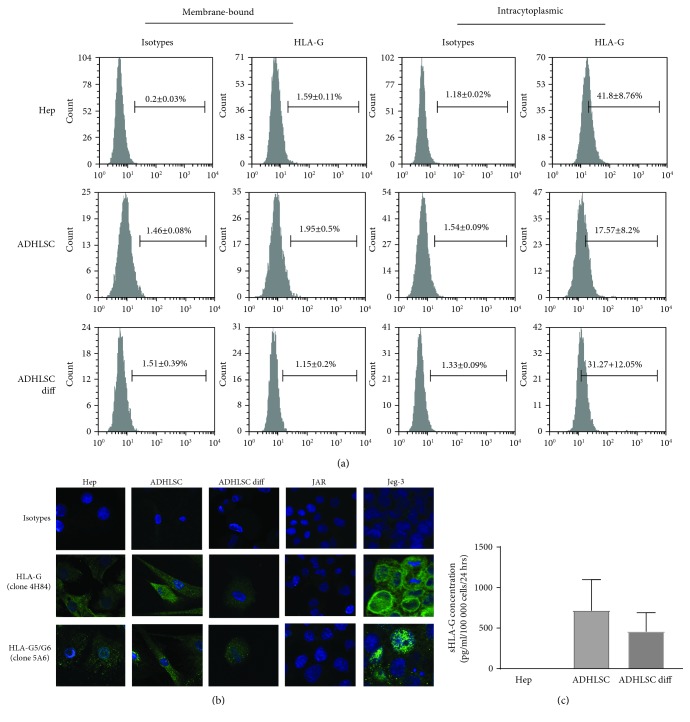
Analysis of HLA-G protein expression by human liver cells. (a) The membrane and intracytoplasmic expression of HLA-G by hepatocytes (Hep), undifferentiated ADHLSCs (ADHLSCs), and differentiated ADHLSCs (ADHLSCs diff) was evaluated by flow cytometry using PE-anti-HLA-G1/-G5 IgG1 (clone MEM-G9, which recognizes both membrane-bound HLA-G1 and soluble HLA-G5/-G6 isoforms). Gates were placed based on the corresponding isotype. Results in percent of positive cells are expressed as mean ± SEM. The images shown are representative of at least 3 independent experiments. (b) The expression of HLA-G by human liver cells was determined by immunofluorescence. Cells were stained with specific antibodies (clone 4H84 or 5A6 which recognize all HLA-G isoforms or HLA-G5 and HLA-G6 isoforms, respectively) or the corresponding isotype control antibodies followed by Alexa Fluor 488-conjugated secondary antibodies (green). The samples were then counterstained with DAPI in order to visualize the nuclei (blue). Picture magnification is 400x. (c) The presence of soluble HLA-G in the culture supernatants of hepatocytes and undifferentiated and differentiated ADHLSCs was evaluated using a Luminex assay. Results are expressed as pg/ml/100 000 cells/24 hours.

We then evaluated HLA-G expression at the protein level. The expression of the membrane and intracytoplasmic HLA-G by human liver cells was analyzed by flow cytometry. As shown in Figure 3(a), intracellular HLA-G was detected in both human hepatocytes and ADHLSCs. After hepatogenic differentiation, HLA-G expression seemed upregulated in ADHLSCs, although significance was not reached due to the high variability. In addition, expression of HLA-G-soluble isoforms was confirmed for hepatocytes and ADHLSCs using fluorescence microscopy (Figure 3(b)). Moreover, sHLA-G was detected in the supernatants of both differentiated ADHLSCs and undifferentiated controls (2 out of 3 samples) but the variability did not allow us to conclude on a potential difference between the two groups (Figure 3(c)). In addition, we did not find sHLA-G in the supernatants of hepatocytes. Finally, flow cytometry analysis of human hepatocytes and ADHLSCs showed no expression of the HLA-G receptors ILT2, ILT4, and KIR2DL4 (data not shown).

#### 3.1.3. Human Hepatocyte and Differentiated Liver Progenitor Cell In Vitro Immune Inhibitory Activities Are Mediated through HLA-G

**Figure 4:**
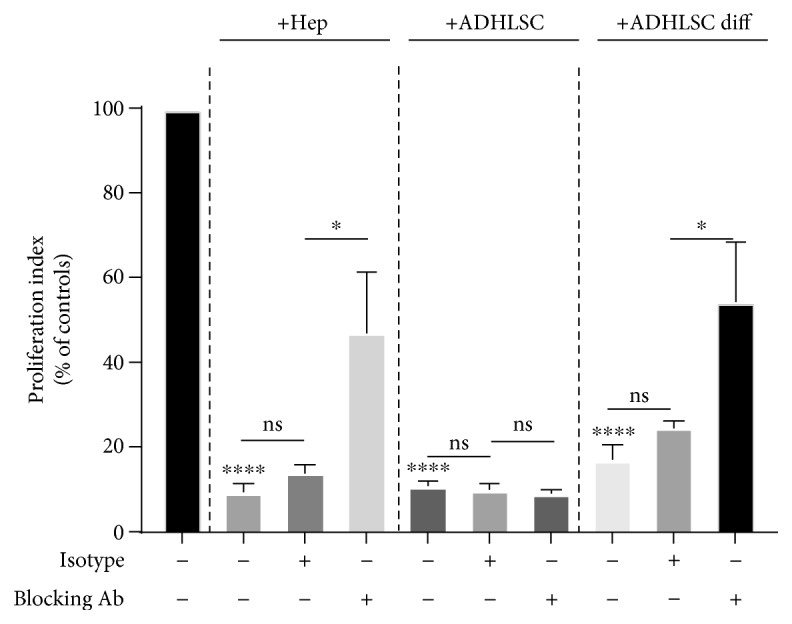
The *in vitro* immune inhibitory properties of liver cells mediated by HLA-G. Hepatocytes, undifferentiated ADHLSCs, or differentiated ADHLSCs were added as third-party cells to MLR cultures at the MLR : cell ratio of 1 : 0.5 for 7 days. Where specified, liver cells were added to the MLR in the presence of neutralizing anti-HLA-G antibody (clone 87G) or isotype control antibody. The proliferation of allogeneic responder PBMCs was evaluated by the [^3^H]-thymidine incorporation method. The proliferation obtained in the MLR was arbitrarily assigned a value of 100% and used as a reference to calculate the percentage of proliferation obtained with the other conditions. The bars represent the mean ± SEM of at least 3 independent experiments. The data were analyzed using the one-way ANOVA test followed by the Tukey post hoc test for multiple comparisons of the MLR group to the other treatment groups. ^∗^*p* ≤ 0.05 and ^∗∗∗∗^*p* ≤ 0.0001.

To assess whether HLA-G expression by liver cells was linked to their *in vitro* immune inhibitory function, hepatocytes or ADHLSCs were added as third-party cells in MLR in the presence or absence of a specific blocking antibody directed against HLA-G. The resulting proliferative response was then measured in a [^3^H]-thymidine incorporation assay. As shown in Figure 4, the immune inhibitory activities of hepatocytes and differentiated ADHLSCs were partially reversed by adding the HLA-G blocking antibody. However, the same phenomenon was not detected with undifferentiated ADHLSCs.

#### 3.1.4. The Upregulation of HLA-G in ADHLSCs following Hepatogenic Differentiation Is Induced by OSM

**Figure 5:**
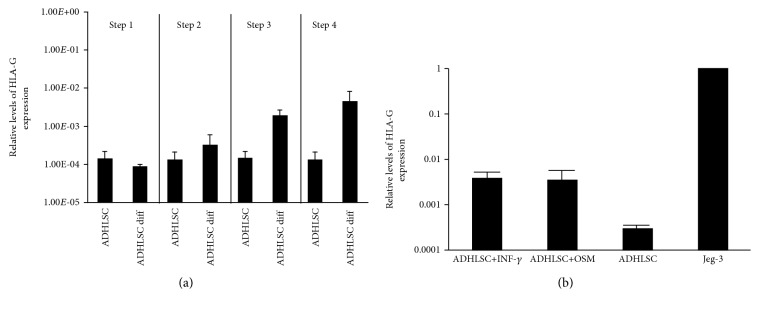
Analysis of HLA-G mRNA expression during hepatogenic differentiation or following OSM treatment. The mRNA expression of HLA-G following each step of the hepatogenic differentiation protocol (a) or after OSM or INF-*γ* treatment (b) was evaluated by real-time RT-PCR and compared to the level of HLA-G mRNA from undifferentiated ADHLSC. The choriocarcinoma cell line Jeg-3 was used as positive control of HLA-G mRNA expression. Results are expressed as the mean ± SEM of three independent experiments and are depicted as relative quantities of HLA-G transcript compared to that found in Jeg-3 (arbitrarily assigned a value of 1).

**Figure 6:**
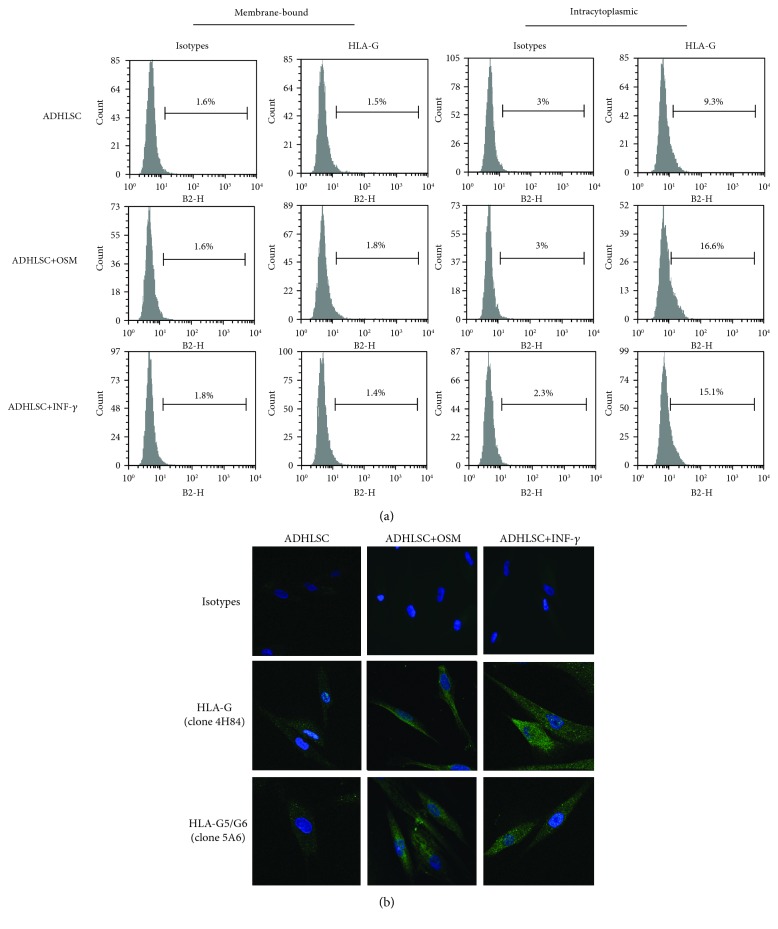
Analysis of HLA-G protein expression following OSM treatment. (a) The membrane and intracytoplasmic expression of HLA-G by ADHLSCs following OSM or INF-*γ* treatment was evaluated by flow cytometry using PE-anti-HLA-G1/-G5 IgG1 (clone MEM-G9, which recognizes both the membrane-bound HLA-G1 and the soluble HLA-G5/-G6 isoforms). Gates were placed based on the corresponding isotype. Results in percent of positive cells are expressed as mean ± SEM. The images shown are representative of at least 3 independent experiments. (b) The protein expression of HLA-G by ADHLSCs after treatment with OSM or INF-*γ* was determined by fluorescence microscopy and compared with ADHLSCs cultured alone. Cells were stained with specific antibodies (clone 4H84 or 5A6 which recognizes all HLA-G isoforms or HLA-G5 and HLA-G6 isoforms, respectively) or the corresponding isotype control antibodies followed by Alexa Fluor 488-conjugated secondary antibodies (green). The samples were then counterstained with DAPI in order to visualize the nuclei (blue). Picture magnification is 400x.

In order to further investigate the upregulation of HLA-G following hepatogenic differentiation, we monitored HLA-G expression at each step of the differentiation process. As shown in Figure 5(a), HLA-G expression remained constant through the first two steps of the hepatogenic differentiation process and increased after the third step, which is characterized by the addition of OSM. To confirm the impact of this growth factor on HLA-G expression, ADHLSCs were cultured in the presence or absence of OSM for 48 hours, using IFN-*γ* treatment as a positive control. Interestingly, a 48 h treatment of ADHLSCs with OSM seemed to induce an increase in HLA-G expression both at the mRNA (Figure 5(b)) and the protein (Figures 6(a) and 6(b)) levels.

#### 3.1.5. Oncostatin M-Induced Upregulation of HLA-G Is Sufficient to Confer Immune Inhibitory Properties to ADHLSCs

**Figure 7:**
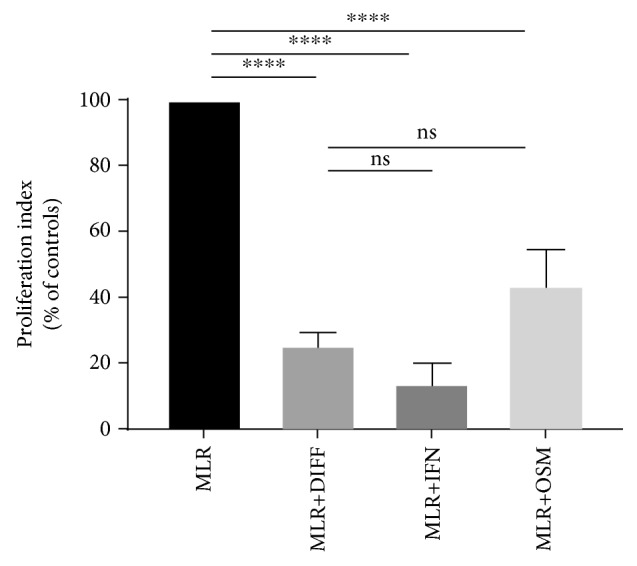
The *in vitro* immune inhibitory properties of liver cells treated with OSM. ADHLSCs previously treated with OSM or IFN-*γ* were added as third-party cells to MLR cultures at the ratio of 1 : 0.5 for 7 days. Differentiated ADHLSCs were used as a control. The proliferation of allogeneic responder PBMCs was evaluated by the [^3^H]-thymidine incorporation method. The proliferation obtained in the MLR was arbitrarily assigned a value of 100% and used as a reference to calculate the percentage of proliferation obtained with the other conditions. The bars represent the mean ± SEM of at least 3 independent experiments. The data were analyzed using the one-way ANOVA test followed by the Tukey post hoc test for multiple comparisons of the MLR group to the other treatment groups. ^∗^*p* ≤ 0.05 and ^∗∗∗∗^*p* ≤ 0.0001.

In order to determine whether OSM alone could confer immune inhibitory properties to ADHLSCs, OSM-treated liver cells were added as third-party cells in MLR and the resulting proliferation was measured using [3]-H thymidine incorporation. As shown in Figure 7, OSM-treated ADHLSCs prevented the alloproliferative response of PBMCs to the same extent as differentiated ADHLSCs.

## 4. Discussion

MSCs in general are known for their immunomodulatory properties. However, the extent of their effect can vary depending on the tissue of origin. ADHLSCs are currently developed as therapeutic medicinal products for the treatment of liver defects. In comparison to mature hepatocytes, the immune profiling of ADHLSCs, both in a basic state or after hepatogenic differentiation, has shown distinct immunoregulatory and immunological marker expression [45, 46]. We have already shown that ADHLSCs present a poor immunogenic profile, which is maintained regardless of their differentiation state, confirming thus their safety for liver cell immune therapy [37, 39, 45]. Here, we demonstrate that hepatocytes and ADHLSCs possess an immune inhibitory activity mediated, at least in part, by HLA-G. In addition, we found that HLA-G-increased expression in ADHLSCs following hepatogenic differentiation is probably due to oncostatin M.

We and others have already demonstrated the poor immunogenicity of human hepatocytes and their ability to induce tolerance, either by inducing tolerogenic DCs or by triggering the death of CD8+ T cells [37, 39, 47–50]. In addition, we have shown that ADHLSCs under normal expansion culture conditions can suppress the proliferative response of T cells to a mitogenic stimulus and that they express HLA-G [35]. However, we have not yet investigated the effect of hepatocytes on T cell proliferation, or the potential link between HLA-G and ADHLSC-induced immunomodulation. In the present study, we showed for the first time that isolated human hepatocytes exhibit an immunosuppressive activity.

Previous studies by Bumgardner et al. have demonstrated a high immunogenicity of infused hepatocytes and an implication of T cells and host APCs in the hepatocyte allograft rejection, but these were performed using murine transplantation models and there is very little evidence of the existence of a similar phenomenon in humans so far [51]. One study by Allen et al. has suggested a T cell-mediated rejection of allogeneic human hepatocytes following liver cell transplantation in one patient with Crigler-Najjar syndrome type I [52]. However, it has to be noted that the CD8+ T cell alloreactivity was evaluated by restimulating recipient PBMCs with irradiated PBMCs or transfected B-lymphoblastoid cell lines (B-LCL) presenting some HLA antigens in common with the donor hepatocytes [52]. This could have an impact on the results because PBMCs and B-LCL are of immune origin and therefore present a different immunogenic potential. In addition, there was no comparison with the response of recipient PBMCs harvested before LCT.

We demonstrated that ADHSLC-mediated immunosuppression was lower than that of hepatocytes but that their potency increased following hepatogenic differentiation. Previous scientific reports demonstrated that BM-MSCs maintained and even increased their capacity to reduce lymphocyte proliferation in MLR after differentiation; however, the authors only considered the chondrogenic, osteogenic, and adipogenic differentiation [53]. Our data suggest that it may be true for hepatogenic differentiation as well.

HLA-G is an immunomodulatory protein, which has been shown to play a role in the immunosuppressive effect of various types of MSCs. Tissue-specific MSCs present a modulated expression of immune regulatory molecules such as HLA-G [41]. We had previously shown using flow cytometry that ADHLSCs do express intracellular HLA-G, consistent with previous reports on BM-MSCs [10]. Here, we show that ADHLSCs express low levels of HLA-G mRNA, which appear to increase after hepatogenic differentiation. In addition, we confirmed the intracellular presence of HLA-G by flow cytometry, which had a tendency to increase following differentiation, and the absence of membrane-bound HLA-G [35, 45]. Further, we demonstrated the presence of soluble forms in both groups by fluorescence microscopy. Finally, we were able to detect sHLA-G in the culture supernatant of both undifferentiated and differentiated ADHLSCs, although there was no statistical difference between the two groups.

HLA-G expression by isolated hepatocytes remains controversial. Indeed, groups have reported its expression at the mRNA level but have failed to demonstrate the presence of the protein [54]. We confirmed the expression of HLA-G at the mRNA level. Further, similar to the results obtained with ADHLSCs, we were able to show the presence of the intracellular form of the protein by flow cytometry and demonstrated that soluble forms were expressed by fluorescence microscopy. We were not able to show the presence of sHLA-G in the culture supernatant of hepatocytes. However, it has to be noted that this is likely due to a high background in the culture medium itself, which was not an issue with the ADHLSC culture medium. In addition, it is well known that hepatocytes do not thrive in culture, which may have affected their production of HLA-G.

The 4H84 clone was used to detect HLA-G expression by fluorescence microscopy, rather than the MEMG-9 clone used for flow cytometry and Luminex assay, because MEMG-9 does not work on fixed slides, which we used in our microscopy analysis. The use of the 4H84 clone has been previously described and validated [55].

In this study, we found that HLA-G expression was increased in ADHLSCs following hepatogenic differentiation. To try and understand the reason for this increase, we looked at HLA-G expression following each step of the differentiation process and showed that HLA-G expression remained constant between steps 1 and 2 but increased following step 3, which is performed with the same medium as step 2 except for the addition of OSM. We then looked at the effect of a 48 hr treatment with OSM alone on HLA-G expression and found that OSM alone was sufficient to induce HLA-G expression. In contrast, we have previously demonstrated a downregulation of CD200 expression in differentiated ADHLSCs after EGF and oncostatin M treatment [45]. In addition, OSM treatment also increased ADHLSC-induced immunosuppression of lymphocyte proliferation. Glucocorticoids such as hydrocortisone and dexamethasone have been shown to increase HLA-G expression in trophoblasts [56]. As dexamethasone is used with OSM in step 4, it may help maintain increased HLA-G expression.

The levels of sHLA-G detected in the culture supernatants of ADHLSCs were low but detectable. Some groups have reported higher levels of HLA-G for other types of MSCs, but often, the exact culture conditions, such as the number of cells and length of the culture, are not described [11, 57, 58]. In addition, the assays used vary in type (ELISA vs Luminex), capture and detection antibodies, and protein source for the standard curve, so a direct comparison is difficult [43, 59]. Bortolotti et al., however, detected between 200 and 260 pg/ml of sHLA-G in supernatants from endometrial tissue MSCs depending on the passage, which seems to be in range with what we found to be produced by 100000 ADHLSCs in 24 hrs, although the culture conditions are not described in their paper either [60].

The pattern of HLA-G expression appeared to follow the immune inhibitory capacity of hepatocytes and ADHLSCs, suggesting a potential link between HLA-G expression and the induction of immune inhibition. By using a blocking antibody directed against HLA-G, we were able to show that HLA-G plays a prominent role in the inhibition of T cell proliferation by both hepatocytes and differentiated ADHLSCs, but not by undifferentiated ADHLSCs, suggesting that other mechanisms may be involved. Several other molecules have been described as playing an important and potentially redundant role in MSC-induced immunomodulation, including IL-10, IDO, and PGE_2_. The secretion of some of these cytokines may be elevated in undifferentiated ADHLSCs and lower following differentiation, which could explain why blocking HLA-G does not have much impact on the immunomodulatory effect of undifferentiated ADHLSCs but does partially reverse the immunomodulatory effect of differentiated ADHLSCs.

Soluble HLA-G can be quantified by ELISA or by bead-based assay (Luminex). Capture and detection antibody pairs available are limited. In addition, the protein used for the standard curve can also vary. In this study, we used a Luminex assay, as described by Rebmann et al. [42, 43]. The advantages of this technique compared to ELISA are a better sensitivity, smaller sample volume requirement, and a shorter procedure. The assay employs a combination of MEMG-9 anti-HLA-G antibody for capture and anti-*β*2-microglobulin for detection because it is one of the most often used combination, the antibodies are available commercially, and MEMG-9 is considered a reliable HLA-G antibody [43]. However, this setup detects both soluble HLA-G5 secreted by the cells and soluble HLA-G1 produced by proteolytic cleavage from the membrane [43]. It is therefore possible that undifferentiated and differentiated ADHLSCs produce similar amounts of sHLA-G overall but that the proportions of HLA-G5 and HLA-G1 are different. In addition, these two isoforms of HLA-G also exist in a *β*2-microglobumin-free form, which cannot be detected by this assay [61]. Finally, HLA-G1 and HLA-G5 can both be found in a mono- or dimeric form, which cannot be distinguished by this Luminex [14]. However, it has been shown that the dimeric forms offer a more accessible binding site for HLA-G receptors, resulting in a higher affinity and a slower dissociation rate, and an increased inhibitory function [62] [63]. It is possible that the HLA-G produced by hepatocytes and differentiated cells is in a dimeric form, while the HLA-G produced by undifferentiated ADHLSCs remains in a monomeric, less efficient form, resulting in the engagement of other immunomodulatory cytokines.

We have previously shown that ADHLSCs secrete immunomodulatory cytokines and in particular a number of anti-inflammatory cytokines [44]. In addition, we have demonstrated that they inhibit the proliferation of hepatic stellate cells, which are responsible for fibrosis, *in vitro* and i*n vivo* [64]. In this paper, we show that they can inhibit the T cell proliferative response to allogeneic stimulation. Together, these results support the potential use of ADHLSCs in the treatment of fibroinflammatory hepatic diseases.

## Figures and Tables

**Table 1:** General characteristics of liver-derived cell donors.

Donor	Sex	Age	Blood type+rhésus	Cause of death	Type of metabolic disease	Cell type
1	M	33	A+	Cerebral hemorrhage		Hepatocytes
2	F	16	A+	Trauma		Progenitor cells
3	M	18	O+	Trauma (fall)		Progenitor cells
4	M	9	A+	Head trauma		Progenitor cells
5	M	11	O+	Cerebral hemorrhage		Progenitor cells
6	F	48	A+	Stroke		Hepatocytes
7	F	37	A+	Cerebral edema		Progenitor cells
8	F	3	A+	CO poisoning		Hepatocytes
9	F	22 months	A+	Living donor	OCT deficiency	Hepatocytes and progenitor cells
10	F	14 months	A+	Living donor	Type 1B glycogenosis	Hepatocytes
11	F	44	A+	Suicide by hanging		Hepatocytes
12	M	3 days	A+	Respiratory		Progenitor cells
13	F	2	O+	Living donor	Refsum	Hepatocytes and progenitor cells
14	M	7 days	O+	Spinal cord damage following traumatic birth		Hepatocytes and progenitor cells
15	M	4 months	O+	Meningitis (nonsystemic)		Hepatocytes and progenitor cells
16	F	6 days	O+	Severe neonatal asphyxia		Hepatocytes and progenitor cells
17	M	23 months	A+	Living donor	Crigler-Najjar syndrome	Hepatocytes
18	F	48	AB+	Cerebral ischemia		Hepatocytes
19	M	33 months	B+	Living donor	Crigler-Najjar syndrome	Hepatocytes

## Data Availability

The data used to support the findings of this study are included within the article, except for the characterization of the ADHLSCs, which can be provided upon request.
